# Typical Hemolytic Uremic Syndrome Secondary to Influenza A in a Pediatric Patient With Complex Comorbidities: A Rare Encounter

**DOI:** 10.7759/cureus.65746

**Published:** 2024-07-30

**Authors:** Muawia Ahmed, Raghad A Alghorayed, Ehab Hanafy, Mohammed Kamal, Yassir MB, Wessam Soliman, Mustafa M Altoonisi

**Affiliations:** 1 Pediatrics, King Salman Armed Forces Hospital, Tabuk, SAU; 2 Prince Sultan Oncology Center, King Salman Armed Forces Hospital, Tabuk, SAU; 3 Pediatric Intensive Care Unit, King Salman Armed Forces Hospital, Tabuk, SAU

**Keywords:** peritoneal dialysis, multisystem inflammatory syndrome, pediatric intensive care, influenza a, hemolytic uremic syndrome

## Abstract

Hemolytic uremic syndrome (HUS) is a severe condition characterized by hemolytic anemia, thrombocytopenia, and acute kidney injury (AKI). It can be atypical, due to complement dysregulation, or typical, primarily linked to bacterial infections, with viral-induced HUS being extremely rare.

We report the case of a six-year-old male who presented with eight days of upper respiratory tract infection symptoms. Initial treatment for tonsillitis was ineffective. He was admitted to the Pediatric Intensive Care Unit (PICU) with severe dehydration, high-grade fever, and AKI, and was initially suspected of having multi-system inflammatory syndrome in children (MIS-C). Further investigation confirmed typical HUS, likely secondary to Influenza A. The child required peritoneal dialysis and other supportive treatments until recovery.

This case underscores the need to consider viral-induced HUS in pediatric patients with severe infections and complex medical presentations. An interdisciplinary approach and timely interventions were crucial for his recovery. This rare presentation of HUS associated with Influenza A highlights the importance of clinical awareness and the need for further research to improve care strategies for similar cases.

## Introduction

Hemolytic uremic syndrome (HUS) is a rare but severe condition predominantly characterized by the triad of hemolytic anemia, thrombocytopenia, and acute kidney injury (AKI). The most common etiology of HUS is infection with Shiga toxin-producing Escherichia coli (STEC), particularly serotype O157. However, atypical HUS can arise from various other triggers, including genetic mutations and infections not related to STEC [1]. Viral-induced HUS, although rare, has been documented in the literature, with cases linked to influenza, HIV, and cytomegalovirus (CMV) [2].

Influenza A, a highly contagious respiratory virus, is known for causing significant morbidity and mortality in pediatric populations, particularly among those with underlying health conditions [3,4]. Complications from Influenza A can range from mild respiratory symptoms to severe outcomes such as pneumonia and acute respiratory distress syndrome (ARDS). In rare instances, Influenza A has been implicated in the onset of HUS, posing diagnostic and therapeutic challenges due to its atypical presentation [5].

The complexity of managing HUS increases significantly when it occurs in pediatric patients with multiple comorbidities. Such cases require a high index of suspicion and a comprehensive, multidisciplinary approach to management. Early recognition and prompt treatment are critical to improving outcomes. This case report describes a unique presentation of HUS secondary to Influenza A in a six-year-old male with a history of glycine encephalopathy, global developmental delay, epilepsy, and thrombocytosis. The aim of this study is to highlight the diagnostic challenges, management strategies, and the importance of considering viral etiologies in the differential diagnosis of HUS in pediatric patients with complex medical backgrounds.

## Case presentation

A.A., a six-year-old male with a history of glycine encephalopathy, global developmental delay, epilepsy, and secondary thrombocytosis, was regularly followed up at neurology and hematology clinics. He presented to our hospital in October 2023 with eight days of upper respiratory tract infection symptoms, including a runny nose, congested throat, and low-grade fever.

On October 21, 2023, the patient visited the ED with decreased oral intake and symptoms of an upper respiratory tract infection, including a subjective fever. Diagnosed with tonsillitis, he was discharged with antibiotics. However, his father reported no improvement. Two days later, he experienced persistent vomiting and significant diarrhea. On October 23, 2023, he returned to the ED with swelling in his right foot and ongoing diarrhea. After thorough investigations yielded reassuring results, the patient was discharged home.

On October 29, 2023, the child returned to the ED concerned about the absence of urine output for one day, no oral intake, and a documented high-grade fever (39°C). He was admitted to the pediatric intensive care unit (PICU) on October 30, 2023, with a clinical impression of septic shock versus hypovolemic shock, AKI, and potential multi-system inflammatory syndrome in children (MIS-C) (Table 1).

**Table 1:** Laboratory work up at admission and upon discharge. CRP: C-Reactive Protein; Hg: Hemoglobin; PT: Prothrombin Time; PTT: Partial Thromboplastin Time; INR: International Normalized Ratio.

	Lab Values at Admission	Lab Values at Discharge
Sodium	131 mmol/l	139 mmol/l
Potassium	7.4 mmol/l	4.2 mmol/l
Chloride	99 mmol/l	109 mmol/l
Bicarbonate	17 mmol/l	22 mmol/l
Urea nitrogen	55 mmol/l	1.9 mmol/l
Creatinine	647 µmol/l	27 µmol/l
Magnesium	0.84 mmol/l	0.69 mmol/l
Phosphate	3.26 mmol/l	1.95 mmol/l
CRP	16.50 mg/L	2.6 mg/L
Calcium	1.79 mmol/l	2.06 mmol/l
Procalcitonin	34.43 ng/mL	3 ng/mL
Total protein	43 g/L	57 g/L
Albumin	24 g/l	33 g/l
WBC	21.15 x 10^3^/µl	18.12 x 10^3^/µl
Hg	7.5 g/dl	9.3 g/dl
Platelets	98 x 10^3^/µl	506 x 10^3^/µl
D-dimer	16.9 mg/ml	1 mg/ml
Fibrinogen	506 mg/dl	240 mg/dl
PT	15.30 sec	11.90 sec
PTT	40.6 sec	40.9 sec
INR	1.11	0.85
Reticulocytes	1.4%	1.3%
Amylase	744 u/l	340 u/l
Lipase	1264 u/l	320 u/l
Lactic acid dehydrogenase	3490 u/l	903 u/l
Respiratory viral panel	Influenza A (positive)	-

Upon admission, the child appeared severely dehydrated, with sunken eyes, prolonged capillary refill time (>4 seconds), tachycardia, and weak peripheral pulses. His heart rate was 118 bpm, respiratory rate 20 breaths/min, blood pressure 120/44 mmHg, and C-reactive protein (CRP) > 4 mg/L. The chest examination revealed decreased bilateral air entry with crepitations, and the abdominal examination was soft and lax without organomegaly. The cardiovascular examination showed normal heart sounds with no murmurs or added sounds.

The child received initial fluid resuscitation and intravenous antibiotics. His electrolyte imbalances, including hyperkalemia and hyponatremia, were managed. Due to low hemoglobin levels, he received a transfusion of packed RBCs (PRBCs). A bedside echocardiogram showed a distended inferior vena cava, prompting fluid restriction and a diuretic infusion. Due to oliguria, urgent peritoneal dialysis was initiated. Post-procedure, he became hypotensive and required inotropic support. He was kept intubated on mechanical ventilation.

After two days, a viral panel revealed Influenza A positivity. The child's multidisciplinary team initially suspected MIS-C, and he received intravenous immunoglobulin (IVIG). However, his clinical picture, combined with a decrease in hemoglobin and platelets (which were consumed shortly after transfusion), high lactic acid dehydrogenase (LDH) levels, and the presence of significant schistocytes on the peripheral blood film, led to a final diagnosis of typical HUS, likely secondary to the Influenza A infection.

The patient was managed as having typical HUS, and further investigations were conducted to identify the underlying cause of the infection. However, all bacterial cultures were negative, and only the viral screening showed Influenza A.

Following the first dialysis session, there was significant improvement in pulmonary function, a reduction in generalized edema, and improved myocardial function. Stool cultures were not taken, as the child did not pass stool during his PICU stay. Two days post-IVIG, he showed clinical improvement, was extubated, and transitioned from bilevel positive airway pressure (BiPAP) to a nasal cannula, eventually breathing room air. He continued peritoneal dialysis with gradual improvement in urine output. Inotropic support was discontinued, and his thrombotic profile improved. Nasogastric tube feeding was started, and the total antibiotic course was completed.

A neurology consultation was performed to review his medication due to persistent tremors. Adjustments in his medication successfully resolved the tremors, and the child returned to his baseline neurological status. Daily dialysis was gradually tapered off and discontinued after 11 days. The peritoneal dialysis line was removed after three weeks of hospital stay.

The patient was discharged in a clinically stable condition, having returned to baseline activity with good oral intake and acceptable urine output.

## Discussion

HUS is a clinical syndrome characterized by a triad of microangiopathic hemolytic anemia, thrombocytopenia, and AKI [6]. The pathogenesis involves endothelial cell damage, leading to platelet activation and aggregation, RBC fragmentation, and impaired renal function. The most common cause of HUS in children is infection with STEC, particularly serotype O157 [1]. These cases, known as typical HUS, are usually associated with gastrointestinal infections that result in bloody diarrhea. HUS can also arise from other triggers, including genetic mutations, complement dysregulation, and non-STEC infections, which are classified as atypical HUS [2].

The management of HUS primarily involves supportive care, focusing on maintaining fluid and electrolyte balance, controlling hypertension, and providing renal support as necessary. In severe cases, renal replacement therapy, such as dialysis, may be required due to significant renal impairment [3]. Blood transfusions are often administered to manage severe anemia, and platelet transfusions are considered for significant bleeding. In cases of atypical HUS, additional interventions such as plasma exchange or complement inhibition therapy with agents like eculizumab may be necessary [7].

In this report, we present a rare case of typical HUS secondary to Influenza A infection in a six-year-old male with significant comorbidities, including glycine encephalopathy, global developmental delay, epilepsy, and secondary thrombocytosis. The patient initially presented with symptoms of an upper respiratory tract infection and was later diagnosed with HUS based on clinical findings and peripheral blood film results. This case is particularly notable due to the rarity of HUS being triggered by Influenza A, which typically does not feature prominently in the etiological spectrum of HUS [8].

While bacterial infections are the predominant cause of HUS, viral infections have occasionally been implicated. Influenza-associated HUS is extremely rare, with only a few cases reported in the literature. A study by Silecchia V et al. described a case of HUS following an Influenza A infection, where the patient developed severe hemolytic anemia and acute renal failure, similar to our case [9]. Another report by Watanabe T discussed cases of HUS secondary to Influenza, emphasizing the need for high clinical suspicion and timely intervention in such cases [10]. These instances highlight the necessity for healthcare providers to consider viral etiologies when faced with atypical presentations of HUS.

These cases have been linked to mutations in complement regulation factors and the absence of genetic predispositions, suggesting that the virus may act as a trigger. In vitro and in vivo studies have hypothesized the direct pathogenicity of the H1N1 virus, demonstrating its ability to induce endothelial cell apoptosis, platelet activation, and microthrombi formation. The pathogenicity of the influenza virus also hinges on its capacity to produce neuraminidase (NA), a mechanism shared with Streptococcus pneumoniae [11,12].

Beyond Influenza A, other viral infections have been associated with HUS, though these are rare occurrences. For instance, cases of HUS linked to HIV and CMV have been documented. A study by Badesha showed that HIV infection is linked to various renal disorders, including thrombotic thrombocytopenic purpura (TTP) and HUS in patients with advanced AIDS. The study described a patient who initially presented with HUS, leading to the subsequent diagnosis of HIV infection during the investigation. It emphasizes the importance of suspecting HIV in patients who present with microangiopathic renal failure [13]. Similarly, a study by Waiser J et al. reported the de novo occurrence of HUS immediately following the onset of primary CMV infection in two renal allograft recipients, further broadening the spectrum of viral-induced HUS [14]. These cases underscore the diverse etiologies of HUS and the importance of a thorough diagnostic evaluation in atypical presentations.

The management of HUS, particularly in complex cases involving multiple comorbidities, necessitates a multidisciplinary approach. In our case, the patient's care was coordinated among various specialists, including pediatric nephrologists, intensivists, hematologists, and neurologists. The initial management focused on stabilizing the patient's hemodynamic status through fluid resuscitation and electrolyte correction. Given the severity of the patient's condition, urgent peritoneal dialysis was initiated to manage AKI and fluid overload. Additionally, IVIG therapy was administered, suspecting an inflammatory or immune-mediated component to the patient's HUS [15].

The involvement of a multidisciplinary team ensured comprehensive care, addressing not only the renal and hematologic aspects of HUS but also the patient's neurological and overall health status. Regular consultations and coordinated care plans were crucial in managing the patient's complex clinical picture, leading to significant clinical improvement and eventual recovery.

## Conclusions

Typical HUS can be induced by both viral and bacterial infections, with Influenza A being an extremely rare cause. This case highlights the complexity of managing a pediatric patient with multiple comorbidities and severe infection. An interdisciplinary approach and timely interventions, including peritoneal dialysis and IVIG, were crucial to the patient's recovery. The diagnosis of HUS, secondary to Influenza A, underscores the importance of considering this rare etiology in similar cases. Further research and case reporting are vital to optimize care for pediatric patients with complex medical conditions.
